# Smartphone apps for calculating insulin dose: a systematic assessment

**DOI:** 10.1186/s12916-015-0314-7

**Published:** 2015-05-06

**Authors:** Kit Huckvale, Samanta Adomaviciute, José Tomás Prieto, Melvin Khee-Shing Leow, Josip Car

**Affiliations:** Global eHealth Unit, Imperial College London, Reynolds Building, St Dunstans Road, London, W6 8RP UK; CRG, École polytechnique, Palaiseau, France; Department of Endocrinology, Tan Tock Seng Hospital, Singapore, Singapore; Office of Clinical Sciences, Duke-NUS Graduate Medical School, Singapore, Singapore; Singapore Institute for Clinical Studies, A*STAR, Singapore, Singapore; Health Services and Outcomes Research Programme, LKC Medicine, Imperial College – Nanyang Technological University, Singapore, Singapore

**Keywords:** app, Smartphone, Mobile health, mHealth, Calculator, Diabetes, Safety

## Abstract

**Background:**

Medical apps are widely available, increasingly used by patients and clinicians, and are being actively promoted for use in routine care. However, there is little systematic evidence exploring possible risks associated with apps intended for patient use. Because self-medication errors are a recognized source of avoidable harm, apps that affect medication use, such as dose calculators, deserve particular scrutiny. We explored the accuracy and clinical suitability of apps for calculating medication doses, focusing on insulin calculators for patients with diabetes as a representative use for a prevalent long-term condition.

**Methods:**

We performed a systematic assessment of all English-language rapid/short-acting insulin dose calculators available for iOS and Android.

**Results:**

Searches identified 46 calculators that performed simple mathematical operations using planned carbohydrate intake and measured blood glucose. While 59% (n = 27/46) of apps included a clinical disclaimer, only 30% (n = 14/46) documented the calculation formula. 91% (n = 42/46) lacked numeric input validation, 59% (n = 27/46) allowed calculation when one or more values were missing, 48% (n = 22/46) used ambiguous terminology, 9% (n = 4/46) did not use adequate numeric precision and 4% (n = 2/46) did not store parameters faithfully. 67% (n = 31/46) of apps carried a risk of inappropriate output dose recommendation that either violated basic clinical assumptions (48%, n = 22/46) or did not match a stated formula (14%, n = 3/21) or correctly update in response to changing user inputs (37%, n = 17/46). Only one app, for iOS, was issue-free according to our criteria. No significant differences were observed in issue prevalence by payment model or platform.

**Conclusions:**

The majority of insulin dose calculator apps provide no protection against, and may actively contribute to, incorrect or inappropriate dose recommendations that put current users at risk of both catastrophic overdose and more subtle harms resulting from suboptimal glucose control. Healthcare professionals should exercise substantial caution in recommending unregulated dose calculators to patients and address app safety as part of self-management education. The prevalence of errors attributable to incorrect interpretation of medical principles underlines the importance of clinical input during app design. Systemic issues affecting the safety and suitability of higher-risk apps may require coordinated surveillance and action at national and international levels involving regulators, health agencies and app stores.

**Electronic supplementary material:**

The online version of this article (doi:10.1186/s12916-015-0314-7) contains supplementary material, which is available to authorized users.

## Background

The rise of apps, software programs that run on devices like smartphones, creates novel opportunities for healthcare and disease management. Consumer surveys suggest that three-fifths of US adults [1], and over 1.5 billion worldwide [2], own an app-capable smartphone. App stores are populated with increasing numbers of low-cost or free information apps, diaries, and other tools intended to help individuals manage their health and share data with professionals [3]. Utility functions, such as drug dose calculators, that can be integrated into daily routines have become a common feature in medical apps [3]. While a quarter of adults report already using a health or fitness-tracking app [4], nine out of ten state that they would be happy to receive a mobile app “on prescription” from a physician [5], and a third of clinicians report having recommended an app to a patient in the past year [6]. Apps are now a routine feature of health policy discussions [7-9] and government strategy [10]. While recognizing potential benefits for care experience, outcomes, and resource utilization, recent discussion has identified gaps in evidence concerning effectiveness and safety [11-14].

Medical apps that are poorly designed, or do not function as intended, pose a potential risk to patients and threaten confidence among clinicians, patients, and the broader public. There is a spectrum of risk. Apps that support education and disease tracking may not adhere to evidence-based practices [15-17], but are unlikely to be actively harmful. In contrast, problems in apps with diagnostic and therapeutic functions, including calculators intended to recommend a dose of medication, may directly affect health outcomes. Poor diagnostic sensitivity has been identified in apps offering diagnostic screening for skin cancer [18], raising the possibility of missed or delayed diagnosis. Errors have been found in calculator apps intended to establish treatment thresholds for asthma [19] with potential consequences ranging from unnecessary treatment to unplanned care use resulting from the failure to recognize early signs of deterioration. Medication-related functions deserve particular scrutiny. Adverse drug events resulting from prescriber and patient errors are a common cause of avoidable harm in hospital and ambulatory care [20-23]. Up to one in seven patients report having experienced a medication error in the past two years [24]. The potential for new risks arising from the use of medication apps is suggested by the withdrawals of a small number of products, including an insulin dose calculator developed by a pharmaceutical company, because of clinically relevant errors [8,25]. While calculator apps designed for use by healthcare professionals have been subject to scrutiny [26], the safety characteristics of those intended for patient use are largely unknown.

This study aimed to systematically explore the clinical suitability of app-based dose calculators intended for patient use, using the example of diabetes mellitus as a long-term condition that is routinely encountered in ambulatory and hospital care. Many patients with diabetes need to adjust long-acting therapy with a series of tailored insulin doses taken throughout each day before meals and snacks, or to correct high blood sugar levels. Individuals must calculate a dose that correctly integrates expected carbohydrate intake, measured blood glucose levels, and past insulin doses [27]. To assist with this process, dedicated dose calculators are already available, both as dedicated physical devices and integrated into pumps and meters. Apps offer a novel platform for calculation that is available wherever a patient takes his or her smartphone. We undertook a systematic assessment of the design and behavior of English-language rapid-acting insulin calculators. We attempted to provide an exhaustive characterization of issues that might affect their suitability for use in clinical practice.

## Methods

The study used a cross-sectional design which attempted to review all apps available at a particular point in time. We adapted principles from systematic literature review to structure a process of app identification and evaluation that aimed to minimize bias and error during assessment.

### App selection

The UK versions of online app marketplaces for the two most widely used smartphone operating systems were searched in August 2013 using a predefined strategy (detailed in Additional file 1: Methods AF1). Obviously irrelevant apps were eliminated by reviewing app store descriptions and screenshots to identify apps that were either unrelated to diabetes self-management or for which no calculator could be present, for example, diabetes eBooks. The remaining apps were downloaded and evaluated using prespecified inclusion and exclusion criteria. Each stage of search and selection was performed by at least two investigators (of KH, SA, and JTP), working independently. Consensus was reached by pooling independent decisions and resolving any discrepancies through group discussion involving all search investigators in which discrepant apps were re-reviewed against selection criteria. We planned to involve an independent arbiter (JC) in the event that resolution was not possible, but in practice this was not needed.

Apps were eligible for inclusion if they were in the English language and contained a tool for performing a mathematical operation on one or more patient- and context-specific numeric parameters to suggest a dose of rapid or short-acting insulin. We applied the following inclusion and exclusion criteria:

Inclusion criteria

- Smartphone or tablet app
- Containing a rapid-acting insulin dose calculator, a tool to perform a mathematical operation on one or more patient- and context-specific numeric parameters
- English language
- Targeted at patients of any age or clinicians, or both (in practice, all the apps included in this study targeted patients)
- Free and paid apps

Exclusion criteria

- Not available through Android or iOS device marketplaces
- Could not be downloaded because of country restrictions that prevented access in the United Kingdom
- Could not be used because of technical problems, after two attempts
- Sliding scales, lookup tables or other standard instruments that do not involve mathematical transformation of patient-supplied inputs

Apps were not restricted by cost, country of origin, target platform, download count, or intended audience. Consistent with the approach taken by medical regulators [28], calculators explicitly disclaiming medical use for self-management or treatment were not excluded, since the only valid use of a product advertised as a drug dose calculator is for medical purposes. Apps meeting the inclusion criteria were excluded only if technical issues prevented them from being downloaded or run on test devices. Duplicate apps running on the same platform were excluded. However, apps available on both iOS and Android were retained to assess consistency between the operating systems. App stores were rechecked in October 2014 to see if included apps remained available for download or had been updated during the study period. Updated apps were reevaluated.

### Assessment procedures

Apps were assessed using a standardized method to examine each component of the calculation process (Additional file 1: Methods AF2). Expected inputs and outputs, supported unit systems, terminology, and any supplementary app features were characterized by inspection. Simulated data were used to define behavior in response to missing and extreme input values. Where the formula used for calculation was not displayed in the app or in associated documentation, the developer was contacted. For those apps where a formula was ultimately identified, performance was assessed using a set of test cases generated by permutation of the range of possible values for each input parameter (further described in Additional file 1: Methods AF3). Where present, clinical disclaimer text was extracted and coded to identify statements advocating discussion with a healthcare professional prior to calculator use and the role of personal judgment in interpreting generated results. All apps were assessed by a clinician-researcher (KH) and a second reviewer (either JTP, a mobile health researcher, or SA, a public health researcher), working independently. While also intended to minimize the risk of bias, comparison of these independent reviews served mainly to identify accidental errors or omissions during assessment. We planned for a third investigator to provide arbitration in the event that differences could not be resolved by discussion, but in practice this was not required, and the small number of scoring differences were resolved by rechecking the affected apps.

### Statistical analysis

Issues identified within apps were aggregated for analysis. The schema used for grouping (Table 1) was informed by previous work [19,29] and refined through discussion and divided issues into two broad categories depending on whether they concerned the process of data entry (input issues) or the results that were generated (output issues, Table 1). This partitioning accounted for both the differing potential for error, in our view, and the availability of potentially mitigating strategies. Thus, while input issues tended only to increase the likelihood of an incorrect value being used for calculation, output issues were concerned with errors arising despite no error on the part of a user. Operational criteria for this schema are provided in Additional file 1: Methods AF4. Simple descriptive statistics were used to summarize the prevalence of issues within the schema.

**Table 1:** **Frequency of identified issues, grouped by platform and cost**

			**Frequency by platform (%)**			**Frequency by cost (%)**		
	**Comment**	**All apps (n = 46)**	**Android (n = 21)**	**iOS (n = 25)**	***P*** value*	**Free (n = 21)**	**Paid (n = 25)**	***P*** value*
**Input issues**		44 (96%)	21 (100%)	23 (92%)	0.493	20 (95%)	24 (96%)	1.000
Numeric validation lacking	Inputs unconstrained by physiologically or logically plausible limits, for example, negative values accepted.	42 (91%)	21 (100%)	21 (84%)		20 (95%)	22 (88%)	
No validation of any input	For example, textual values allowed in all numeric fields.	24 (52%)	14 (67%)	10 (40%)		12 (57%)	12 (48%)	
Some validation	Inconsistency reflecting use of validation for only some inputs.	18 (39%)	7 (33%)	11 (44%)		8 (38%)	10 (40%)	
Calculation despite missing inputs	For example, correction bolus calculated even though insulin to blood glucose ratio not provided.	27 (59%)	14 (67%)	13 (52%)		10 (48%)	17 (68%)	
Ambiguous terminology	Use of ambiguous labeling or descriptions creates risk of misinterpretation.	22 (48%)	8 (38%)	14 (56%)		8 (38%)	14 (56%)	
Data entry issues	User data cannot be entered faithfully or are not stored correctly for calculation.	6 (13%)	3 (14%)	3 (12%)		4 (19%)	2 (8%)	
Precision issues	For example, a data field for measurements in mmol/L that accepts only whole integers.	4 (9%)	1 (4%)	3 (14%)		4 (19%)	0 (0%)	
Data not stored correctly	Data change unexpectedly, for example, transposed into a different data field.	2 (4%)	2 (8%)	0 (0%)		0 (0%)	2 (8%)	
**Output issues**		31 (67%)	17 (81%)	14 (56%)	0.115	14 (67%)	17 (68%)	1.000
Clinical model violation	Calculation does not conform to principles of underlying clinical conceptual model, for example, by assuming an omitted blood glucose implies a measured value of zero.	22 (48%)	13 (62%)	9 (36%)		10 (48%)	12 (48%)	
Formula inconsistency †	Calculation does not proceed in accordance with the stated formula.	3 (14%)	1 (11%)	2 (17%)		0 (0%)	3 (23%)	
Input–output mismatch	Calculator output is not consistently synchronized with changing user inputs.	17 (37%)	9 (43%)	8 (32%)		8 (38%)	9 (36%)	
Automatic calculator	A calculator which normally refreshes output automatically in response to changing inputs does not under certain circumstances.	3 (7%)	2 (10%)	1 (4%)		0 (0%)	3 (12%)	
Manual calculator	App does not respond reliably to manual commands to recalculate dose, for example, tapping a “Calculate” button.	14 (30%)	7 (33%)	7 (28%)		8 (38%)	6 (24%)	
**Other software errors**	Software unexpectedly crashes or becomes unresponsive during normal use.	11 (24%)	8 (38%)	3 (12%)	-	4 (19%)	7 (28%)	-

*Two-tailed *P* value for the comparisons between Android and iOS and Free and Paid apps respectively, calculated using Fisher’s exact test.

† The denominator used for calculating percentages for this issue was the number of apps for which a formula was identified (n = 21, Android 9, iOS 12, Free 8, Paid 13).

Input issues affect the entry of data and increase the risk that calculation will proceed without correct parameters. Output issues affect interpretation of the calculated value. They create a risk of incorrect dose recommendation even when all input parameters have been supplied correctly. While input issues may be amenable to corrective action taken by a user, for example, by double-checking data entry, output issues may only be effectively addressed through changes to the software.

Before starting analysis, we hypothesized that the prevalence of identified issues might vary both by platform and the payment model (free or paid), because apps for iOS are subject to a quality control process prior to release whereas Android has no such requirement, and because developers of paid-for apps may invest more in quality assurance. Fisher’s exact test was used to calculate the two-tailed probability of an association between issue prevalence and platform or distribution model. A significance level of 0.05 was prespecified. Statistics were computed with R (Version 3.0.0) using the package exact2x2 (Version 1.4.0).

## Results

**Figure 1:**
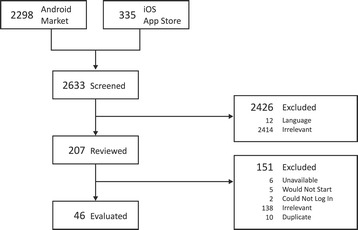
App selection process.

Forty-six apps (21 Android, 25 iOS) were selected for inclusion into the study (Figure 1). Of these, nine were available on both platforms but evaluated separately. Details of excluded apps are provided in Additional file 2: Table AF5.

Approximately two-fifths (37%, n = 17/46) of apps were standalone calculators (Table 2, detailed in Additional file 2: Table AF6). Most (59%, n = 27/46), however, combined a calculator with a diary for recording blood glucose measurements. Over half of apps (59%, n = 27/46) included a clinical disclaimer. Most disclaimers emphasized the importance of discussing use of a calculator with a physician (n = 12/27) and using judgment before accepting any dose recommendation (n = 10/27). A small number (n = 4/27) explicitly stated that the app should not be used for treatment or other medical purposes. However, because these apps were clearly advertised as dose calculators they were retained for analysis. One iOS app was associated with an evidence-based diabetes self-management education program [30,31] and another, available for both iOS and Android, had been developed by healthcare professionals [32]. Download counts were available for Android, but not iOS. While not necessarily reflecting the number of active users, they allow a conservative aggregate estimate of at least 105,000 global downloads of the calculators included in the study by October 2014. While the majority of Android calculators had relatively low download counts (median 1000), three apps had been downloaded at least 20,000 times each.

**Table 2:** **Characteristics of included dose calculator apps**

	**Number by platform (%)**	
	**Android (n = 21)**	**iOS (n = 25)**
**App type**		
Standalone calculator	6 (29%)	11 (44%)
Diary with integrated calculator	14 (67%)	13 (52%)
Other*	1 (5%)	1 (4%)
**Region of origin**		
Europe	11 (52%)	12 (48%)
North America	6 (29%)	8 (32%)
Asia and Australasia	4 (19%)	5 (20%)
**Cost**		
Free	9 (43%)	10 (40%)
Paid for	12 (57%)	15 (60%)
**Releases**		
Updated during study period	5 (24%)	12 (48%)
Updated in 12 months prior	13 (62%)	16 (64%)
Still available at close of study	20 (95%)	22 (88%)
**Self-management features**		
Food database	3 (14%)	5 (20%)
Data sharing	11 (52%)	8 (32%)
Reminders	3 (14%)	3 (12%)
**Other features**		
Clinical disclaimer	11 (52%)	16 (64%)
Accreditation	1 (5%)	2 (7%)
In-app help	12 (57%)	13 (52%)
Support contact	10 (48%)	16 (64%)

*One app available for both Android and iOS contained information about insulin pump therapy but included a dose calculator as an additional feature. Many pumps themselves contain dose calculators, potentially rendering a separate calculator redundant.

All apps performed a basic meal bolus calculation, designed to work out the amount of insulin required to offset counted carbohydrate intake that was extended in most cases (83%, n = 38/46) with an adjustment for current blood glucose measurements (Additional file 2: Table AF7). All apps accepted inputs entered manually by the user. Eight apps provided a food database for quantifying carbohydrate intake. One (Glucose Meter for Android [33]) supported automatic transfer of blood glucose measurements from a Bluetooth-enabled meter. Apps extended the basic calculation by incorporating additional adjustments for exercise levels (n = 10), measured ketones (n = 2), and lipids (n = 1). Two-fifths of apps (39%, n = 18/46) allowed calculations to be tailored by specific events (for example, mealtimes, n = 12), time of day (n = 6), or both (n = 1). No apps performed adjustments for basal insulin regimes or, for type 2 diabetic patients, combined oral and injected therapy.

The formula used to suggest an insulin dose was listed in a third (n = 14/46) of apps and obtained on request for a further seven. Just under half (46%, n = 21/46) of developers did not respond to requests for information, contact details were not found for two, and two refused on grounds of commercial confidence. Formulae were broadly consistent across apps. Meal boluses were generated by dividing carbohydrate intake by a user-supplied, personal carbohydrate factor; the number of grams of carbohydrate required to offset the reduction in blood glucose caused by one unit of rapid-acting insulin. Correction boluses were derived similarly, by dividing the difference between current and target blood glucose values by an insulin sensitivity factor; the expected reduction in blood glucose in response to 1 unit of insulin for an individual. Some apps used the inverse form of carbohydrate (n = 9) and insulin sensitivity (n = 2) factors, performing a multiplication operation to calculate meal and correction boluses instead. Postprandial adjustments of correction boluses were supported in a fifth (22%, n = 10/46) of apps that either attempted to model residual insulin activity (n = 5) or supported post-meal custom factors (n = 5). One app provided a warning that two-hour postprandial corrections should be adjusted to reflect the pharmacokinetics of a recent mealtime dose. Most (76%, n = 35/46), however, relied on the user to know that an adjustment would be required for insulin-on-board to avoid a risk of overdose.

### Input issues

All Android apps (n = 21) and most (92%, n = 23/25) iOS apps had issues affecting data input that might increase the risk of an incorrect value being used for calculation (Table 1). The majority (n = 21/21 and n = 21/25 of Android and iOS apps, respectively) did not apply validation to all numeric inputs. Measured blood glucose was the most likely to have some form of validation. 24% (n = 11/46) of apps either flagged extreme values with a written message (n = 7) or using color (n = 4), but only one refused to complete calculation. Two-fifths (40%, n = 10/25) of iOS and two-thirds (67%, n = 14/21) of Android apps placed no range limits on any input. A small number of apps (n = 1 and n = 4 of Android and iOS, respectively) allowed textual input in numeric fields. Four apps for iOS applied validation to all numeric inputs. Three-fifths (59%, n = 27/46) of all apps allowed calculation to proceed when one or more values was missing. A small number of apps had data entry issues, such as mmol/L glucose measurements constrained to be whole integer values, that meant that data were either not preserved correctly (n = 2) or could not be entered with appropriate precision (n = 4).

Just under half (48%, n = 22/46) of apps used ambiguous terminology to label and describe inputs. Non-specific terms like “correction factor” were common but used inconsistently, describing both insulin sensitivity and carbohydrate factors in different apps. In addition, there appeared to be a geographical basis for certain calculation conventions. Apps originating from the USA (n = 13) expected meal boluses to be calculated using a carbohydrate factor, while most apps from Germany (n = 5/6) required the inverse form of the factor. Poor documentation meant that the expected form would be invisible to users in half (50%, n = 23/46) of apps. In a small number of cases (n = 3), labeling was frankly incorrect. For example, one app used the phrase “Insulin units to decrease blood glucose by 1 mmol/L” to describe insulin sensitivity factor.

### Output issues

When no missing or out-of-range input values were present, the majority of apps with a known formula (86%, n = 18/21) calculated a result consistent with expectations during systematic testing. Only three apps deviated from their stated formulae. Two introduced an undocumented conversion of glucose values expressed in mmol/L into mg/d, an unnecessary step because a patient using SI base units for glucose would almost certainly not have adjustment factors expressed using conventional US units. A third app silently discounted any negative correction bolus, contradicting the stated formula and creating a risk of over-estimating insulin requirements.

Formula concordance did not guarantee, however, that apps would produce correct output under all circumstances. Over two-thirds (67%, n = 31/46) of all apps, including 19 of those with known formulae, carried the risk of displaying an incorrect or inappropriate result despite inputs being configured correctly. Two apps set limits on the maximum insulin dose that would be prescribed, and two used coloring to highlight doses exceeding 20 units, but most apps placed no limits on the calculated insulin dose. Three-fifths (62%, n = 13/21) of Android and two-fifths (36%, n = 9/25) of iOS apps violated basic clinical assumptions, for example, treating a missing glucose measurement as a real valued number of zero for calculation (n = 13) or failing to reduce a recommended meal bolus when blood glucose was below target values (n = 6). Of the 27 apps that performed calculation automatically whenever inputs were changed, three did not always update their output in response to changing inputs. Three-quarters (74%, n = 14/19) of the remaining apps that required a manual button press to perform calculation did not clear the calculated output when inputs were changed.

Challenging our hypotheses, there was no significant difference in the frequency of input or output issues grouped either by platform type or distribution model, although there was a tendency towards a greater proportion of output issues, and clinical model violations in particular, in apps for Android. Only one app, for iOS, was issue-free according to our criteria. A second iOS app had no identified issues, but did not complete systematic output testing because the developer did not respond to requests for details of the formula. If input validation issues were ignored, a further five apps were otherwise issue-free. While comparison of each of the nine app pairs available on both iOS and Android found no differences in expected inputs, formulae, and outputs, inconsistencies were found during quality assessment. In four cases, Android apps performed no numeric validation, while the iOS version either validated some (n = 3) or all (n = 1) fields. In two cases, the iOS version accepted textual values where the Android version did not. In one case, an Android app lacked explanatory text present in the iOS version that prevented misinterpretation of an ambiguously labeled input. A further two Android apps either displayed incorrect behavior when handling missing values (n = 1) or did not permit data entry with appropriate precision (n = 1). At the conclusion of the nine-month review process, 89% (n = 41/46) remained available in app stores. 18 (44%) had been updated, and one known issue (a data entry bug involving values being stored incorrectly) was resolved.

## Discussion

Sustained glycemic control is associated with better outcomes in diabetes [34,35]. Improved control has been observed in patients receiving continuous subcutaneous insulin infusion supplemented with a bolus dose calculator [36,37]. App-based calculators offer a potential alternative for patients using insulin therapy. They may enhance the accuracy of insulin dosing, for example, by helping to precisely quantify carbohydrate intake. By eliminating mental arithmetic, they may reduce the perceived burden of calculation, particularly for those with limited functional numeracy. In addition, a smartphone, an object not typically associated with medical care, may offer a discrete way to determine dosage in social situations like mealtimes. There is certainly interest from patients. At least 105,000 patients worldwide downloaded apps included in this study. Historically these are likely to be patients with type 1 diabetes, for whom insulin therapy is a mainstay. The growing emphasis on tight control in the much larger population of type 2 diabetic patients means that insulin therapy, and the potential user base for calculator apps, is likely to increase in the future.

Appropriately designed apps may also protect against calculation errors. Insulin has a narrow therapeutic range [23], and the incidence of unintentional overdoses relating to medication errors made by patients is increasing as intensive therapy becomes commonplace [38]. While the consequences of insulin overdose are rarely fatal [39], hypoglycemic events are unpleasant and medication-related problems contribute to unscheduled care use by people with diabetes [40]. Our findings show that it is possible to design dose calculation apps that validate user input, generate correct output, apply appropriate dose ceilings, and flag potentially unsafe conditions. However, these apps are in the minority. A lack of standard terminology and simple numeric validation provide latent conditions that increase the risk of unintentional data entry slips and mistakes related to misunderstanding in most apps [41]. While these “input” issues are potentially amenable to intervention, for example, by educating individuals to double-check the data that they enter in an app, we also identified a large number of “output” issues, which reflect fundamental problems in the underlying software that cannot easily be mitigated by users. In particular, two-thirds of included apps carried the risk of inappropriately large outputs being generated when inputs had been specified correctly, including those that would almost certainly result in hypoglycemia. Many apps displayed behaviors that suggested that developers did not fully understand the clinical basis for calculation. For example, there is no situation where it is clinically appropriate to treat an absent blood glucose value as though it is a measured value of zero. Agreement with a documented formula (when available) is an encouraging finding, and one consistent with recent work examining calculator apps for clinicians [26]. However, appropriate insulin dose recommendations must also take into account contextual and behavioral factors. This is particularly important in calculators intended for patients, who may not have sufficient clinical knowledge to be able to reject incorrect doses. Yet over three-quarters of apps did not offer a mechanism for reducing postprandial boluses to reflect residual insulin activity, and one in six did not reduce dose recommendations in response to hypoglycemia. Apps relied exclusively on users to adjust calculation parameters to reflect basal insulin and oral antidiabetic regimes and other factors that might adjust insulin response, like pregnancy.

Detecting potential issues within apps may be challenging. Some issues were only apparent after exhaustive testing. The reasons that might prompt use of a calculator might also make it hard for patients to detect errors during use. Patients with limited numeracy may lack an intuitive basis with which to “sense check” unusual results [42]. The social contexts in which a calculator might be used, such as mealtimes, may mean people attend less to the process of calculation and scrutinizing of the output. App disclaimers frequently encouraged patients to review the calculated dose, but over two-thirds failed to provide details about the underlying formula that would enable this, and only a small number of apps flagged unusual input or output values. One potentially attractive strategy is to equip individuals with skills to recognize inappropriate or potentially harmful app during their initial search. Simple criterion-based approaches have been used to help consumers judge the quality of websites [43], and criteria have recently been proposed for apps that either rely on structured disclosures by developers addressing a number of generic quality domains [44], such as testing undertaken during development, or incorporate proxies for quality, such as disclosure of authorship [45]. Structured reporting has the potential to flag issues that make calculators unsuitable for particular user groups, for example, unit systems incompatible with local practices. However, at present, no calculator-specific instruments exist, while content-independent instruments rely on developers to appreciate that these are relevant concerns in the first place. Approaches that rely on proxies have the potential advantage of simplicity, but require evidence supporting their capacity to predict quality problems [46]. In this study, we found no significant association between price or platform and the prevalence of calculation errors. Further work is needed to explore whether other aspects of content, design, or usability can provide a reliable way for users to anticipate potential quality problems.

For healthcare professionals, there should be substantial caution in actively recommending calculator apps without thorough testing. Clinicians should consider asking about app use as part of routine diabetic review and specifically mention the potential risks associated with calculators when teaching carbohydrate counting, taking the opportunity to promote trusted resources such as dedicated medical app stores [47,48]. Despite the lack of validated approaches, it is unlikely that common sense advice about choosing heath apps would be actively harmful, and clinicians should take advantage of opportunities to promote simple strategies, such as preferentially choosing apps from known, reputable providers until CE-marked or US Food and Drug Administration (FDA)-approved apps become available and learning how to recognize these quality marks. For patients who are unwilling to discontinue use of a calculator app, advice should focus on the importance of double-checking data entry (to address input issues) as well as establishing a ceiling insulin dose above which any dose recommendations should be ignored. If patients are willing, stepping through calculation may be helpful in exposing gross errors. However, it is unlikely that informal testing, even if specifically directed towards exposing the risk of overdose, for example, by simulating implausible hyperglycemia, will uncover all issues. Although the approach described in this study provides a template for robust review, the workload involved should not be underestimated. App updates are frequent, and require both evaluation of new features and confirmation that existing functions still work. One app had introduced an 11-screen configuration process to address the complexity that had arisen as new features were added. Alternative methods, such as expert peer review, may be more efficient but require evidence to support their use in favor of explicit, criterion-based assessment.

If it is infeasible for either patients or clinicians to systematically appraise the safety of health apps, it is also unclear whether coordinated forms of quality assurance will provide rapid redress. In the USA and Europe, where regulatory guidance is now available [28,49], dose calculators almost certainly meet the respective definitions of a medical device and are therefore subject to oversight [50]. However, none of the apps included in this study appear to have completed registration, labeling, or other general controls which are the minimal requirements for products in both regimes. Most remained available in app stores in October 2014, 12 months after the introduction of FDA guidance. Regulators face an unenviable task of balancing risk, regulatory burden and its consequential impact on innovation, and the sheer volume of apps available [8,51,52]. However, allowing higher risk apps, such as calculators, to escape even minimal oversight creates a risk that harms go undetected because apps will escape established mechanisms for surveillance. App accreditation programs are a recent development, independent of government regulators, with a specific focus on clinical quality [48,53,54], but have yet to prove their ability to provide robust assurance, at scale [55].

Previous literature has highlighted the potential for liability and governance problems associated with technologies, such as telehealth, that transcend geographic boundaries [56]. Our findings suggest that apps based on local medical practices may pose a risk when distributed in globalized app stores without regard to whether the same practices operate in other markets. For insulin dose calculation, the consequence of confusing ratio adjustment factors is always overestimation of the required insulin dose. Apps from the USA assume a ratio form that is inverse to that assumed by apps from some European countries. The lack of universal standard terminology, appropriate labeling, or a written formula in most apps means that users have no way of discerning the correct interpretation. It seems likely that this issue, or variations of it, will apply to other kinds of medical apps that are distributed in multiple locations. In October 2014 a diabetes tracking app integrated into Apple’s own Health platform had to be modified after it was discovered that measurements entered in SI units were not being handled correctly [57]. To address localization issues effectively, some form of coordination between regulators and public marketplaces, which control the distribution of most apps, may be necessary.

The study also highlights the importance of development context in understanding the risks that medical apps may pose. App developers responding to enquiries in this study frequently characterized their software as a personal project, often responding to the need of a family member or friend. We found no evidence of developers acting other than in good faith. However, such an approach may be very different from a professional software development context in which medical expertise might be sought and formal quality engineering approaches applied. Personal projects are unlikely to have the resources available to pay for expert guidance, formal testing, or updates as new evidence emerges. Consequently, unlike traditional medical software, users and regulators cannot assume that the app landscape is dominated, or even substantially populated, by products produced by medically competent developers. The findings emphasize the value of due diligence to accompany testing of the product itself when assessing the risk associated with medical apps. When top-down regulation fails to provide robust quality safeguards, consumer behavior and market dynamics might help. The findings of this study provide both clinicians and patients with enough evidence to engage in open and informed exchanges about the appropriateness of medical apps in the particular case of insulin calculators, and future work will likely generate further insights. This bottom-up approach, however, requires individuals to keep up with recent research in mHealth.

The strengths of this study include the exhaustive approach used for sampling and the steps taken to minimize bias and error during assessment. As a cross-sectional analysis, however, the findings reflect issues present at a particular point in time. Both the number of available apps and the issues present within each app will change as apps are released, updated, and withdrawn. Thus, specific findings may have changed, and clinical users should perform their own assessment before recommending, or discouraging users from, a particular app. Future work should examine how issue prevalence changes and whether new issues arise over time. Neither can this study provide evidence of concrete harms resulting from issues identified in the apps that were reviewed. Coordinated programs of surveillance for harms resulting from medical app use, whether run by regulators, accreditation programs, or researchers, should be a priority.

## Conclusions

Despite potential benefits for patients, popular apps offering insulin dose calculation carry a risk of incorrect dose recommendations ranging from those that might lead to suboptimal disease control to those with potentially life-threatening consequences. Healthcare professionals should exercise caution in recommending higher risk tools such as calculators without careful testing, recognizing that a technically accurate calculation is not the same as one that is clinically safe. Given the volume of apps now available, systemic issues affecting the safety and suitability of medical apps may only be addressed satisfactorily through coordinated action at national and international levels involving regulators, health agencies, and app stores. This should seek to address the safety issues that result from global medical app distribution including the need to coordinate the collection of surveillance data about possible harms, ensure that regulation is clear and consistently enforced, and highlight the importance of clinical expertise to developers of medical apps. Otherwise, apps intended for use in chronic disease management and other aspects of healthcare will continue to put patients at avoidable risk of harm.

## Additional files

Additional file 1:
**Supplementary methods AF1-AF4.** Additional details about methods not included in main manuscript. Contents: Methods AF1 - Strategy used to search online app marketplaces; Methods AF2 - Description of standardized assessment method; Methods AF3 - Procedures for generating test cases for assessing calculation accuracy; Methods AF4 - Operational criteria for grouping issues into analytic schema. Additional file 2:
**Supplementary tables AF5-AF7.** Data tables. Contents: Table AF5 - Characteristics of excluded apps; Table AF6 - Basic characteristics of included apps; Table AF7 - Calculator design of included apps.

## Abbreviation

SI: International system of units
