# Supplementary material for: Smartphone apps for calculating insulin dose: a systematic assessment
Source: BMC Med. 2015 May 6;13:106. doi: 10.1186/s12916-015-0314-7 (PMC4433091; doi:10.1186/s12916-015-0314-7)
Supplement: Additional file 1: — Supplementary methods AF1-AF4. Additional details about methods not included in main manuscript. Contents: Methods AF1 - Strategy used to search online app marketplaces; Methods AF2 - Description of standardized assessment method; Methods AF3 - Procedures for generating test cases for assessing calculation accuracy; Methods AF4 - Operational criteria for grouping issues into analytic schema. [file 12916_2015_314_MOESM1_ESM.docx]

# Additional File 1

## Supplementary Methods and Tables

Supplementary Methods AF1

## Strategy used to search online app marketplaces

Supplementary Methods AF2

## Description of standardized assessment method

Supplementary Methods AF3

## Procedures for generating test cases for assessing calculation accuracy

Supplementary Methods AF4

## Operational criteria for grouping issues into analytic schema

Supplementary Methods AF1

## Strategy used to search online app marketplaces

insulin

OR

glucose

OR

bolus

OR

((diabetes OR diabetic OR DM OR BG) AND (dose OR dosing OR calculator OR calc OR algorithm))

Supplementary Methods AF2

## Description of standardized assessment method

Assessment involved a combination of inspection and manual testing. The process was performed by a single investigator, working independently. A structured data extraction form was created to record details in a standard format for subsequent analysis. All results were reviewed by a second investigator. Only issues that could be replicated by the second investigator were retained for analysis.

The assessment process captured the following details:

- Basic app details
  - Platform
  - Cost
  - Version
  - Description and release notes
- Developer information including contact details
- Download statistics (only available for Google Play)
- Input parameters
  - Supported unit systems for glucose and carbohydrate (all other parameters derive from these base measures)
  - Labels used to describe inputs
  - Numeric ranges and precision accepted by app
  - Optional or mandatory status for calculation
  - Methods for entering data
  - Additional configuration options, for example parameters that may be customized by time of day
- Output parameters
  - Labels used to describe outputs
  - Numeric ranges and precision of generated outputs
- Calculation process
  - Process for triggering calculation
  - Impact on calculation of omitting specific parameters
  - Impact on calculation of providing zero-valued parameters
  - Warnings or other messages generated by app
  - Formula used by app, if identified
  - Results of exhaustive testing (see Supplementary Methods S3), if performed
- Details of contact with developer
  - Motivation for developing software
- Other software features
  - Related self-management features, e.g. glucose diary
  - Support contacts
  - Clinical disclaimer
    - Value of seeking professional advice prior to use
    - Role for personal judgment in interpreting results
    - Disclaims all medical uses
  - Third-party accreditation
  - Paid-for enhancements
- Other software issues identified during testing
- Audit trail for testing process

Supplementary Methods AF3

**Procedures for generating test cases for assessing calculation accuracy**

A set of test cases was created for each app where the calculation formula had been identified. Test cases were generated using the following procedure:

1. The full set of input parameters required for calculation were identified through inspection of the formula.
2. For each numeric-valued input parameter:
3. Physiological minimum, typical and maximum values were established using a lookup table (Table AF3.1). This table was pre-populated based on an informal literature review and clinical expertise. Values were selected based on the unit system supported by the app. If the app supported multiple unit systems, values were selected separately for each.
4. Numeric parameter values were then modified by adding a small amount of random noise. Noise was added conservatively such that the adjusted values did not exceed the range defined by the original lookup values.
5. The precision of the resulting values was amended by rounding to the maximum precision supported by the app. In the case where an app allowed the user to customize the precision of input values, values were rounded to 2 decimal places.
6. For binary and categorical parameters (e.g. exercise level) the range of possible values was defined by simply copying the full set of states supported by the app (e.g. none/some/moderate/intensive).
7. For those parameters that were optional for calculation, a ‘null’ value was added to the list of possible values to represent the case when that value was missing. For example, a blood glucose measurement might be reasonably omitted from calculation if a patient has not performed a recent test.
8. In the case where certain parameters could have different values depending on the time of day (for example, insulin sensitivity) we repeated steps 2-4 for each supported time period (e.g. morning/ afternoon/evening). In the case where the user could define their own time periods, we used a minimum of three randomly-constituted time periods spanning a complete 24 hour period.
9. Considering each supported unit system separately, the set of possible values for each parameter were then permuted against each other to generate an exhaustive set of test cases applicable at a particular point in time.
10. For each test case, an expected output was generated by feeding the selected parameter values into the formula. Outputs were rounded to the maximum precision supported by the app.
11. The set of test cases, each consisting a set of input values and an expected output for a particular unit system and time of day (if supported), were written out into a standard form in preparation for testing.

Evaluation proceeded by stepping through each test case. For each, the app was first configured by selecting the appropriate unit system and, if required, setting the time. The pre-specified values were then entered manually into the calculator. The calculation was then triggered and the output compared to the expected value to identify any discrepancies in formula implementation.

Table AF3.1

**Typical and maximum value lookup table for calculation input parameters**

| **Description** | **Units** | **Minimum** | **Typical** | **Maximum** |
| --- | --- | --- | --- | --- |
| Measured blood glucose | mmol/L | 1.0 | 5.6 | 19.4 |
|  | mg/dL | 18.0 | 100 | 350 |
| Blood glucose reduction per unit of insulin  (Insulin sensitivity) | mmol/L/IU | 0.1 | 2.0 | 16.7 |
|  | mg/dL/IU | 1.8 | 36 | 300 |
| Insulin required per unit change in blood glucose | IU/mmol/L | 0.1 | 1.0 | 9.0 |
|  | IU/mg/dL | 0.01 | 0.06 | 0.5 |
| Carbohydrate | grams | 0.0 | 130 | 900 |
|  | carbs | 0.0 | 13.0 | 90.0 |
|  | 10g portions | 0.0 | 13.0 | 90.0 |
|  | 12g portions (bread units) | 0.0 | 10.8 | 75.0 |
|  | 15g portions (exchanges) | 0.0 | 8.7 | 60.0 |
| Carbohydrate intake offset by one unit of insulin (Carbohydrate factor) | grams/IU | 1.0 | 8.0 | 300.0 |
|  | carbs/IU | 0.1 | 0.8 | 30.0 |
|  | 10g portions/IU | 0.1 | 0.8 | 30.0 |
|  | 12g portions (bread units)/IU | 0.1 | 0.7 | 25.0 |
|  | 15g portions (exchanges)/IU | 0.1 | 0.5 | 20.0 |
| Insulin required per unit of carbohydrate | IU/grams | 0.01 | 0.1 | 1.0 |
|  | IU/carbs | 0.1 | 1.0 | 10.0 |
|  | IU/10g portions | 0.1 | 1.0 | 10.0 |
|  | IU/12g portions (bread units) | 0.1 | 1.2 | 12.0 |
|  | IU/15g portions (exchanges) | 0.1 | 1.5 | 15.0 |

Supplementary Methods AF4

**Operational criteria for grouping issues into analytic schema**

| **Issue type** | **Criteria** | |
| --- | --- | --- |
| Input issues |  |  |
| Numeric validation lacking | 1a | No upper bound placed on input values |
|  | 1b | Negative input values accepted |
|  | 1c | Textual input values accepted |
| Calculation despite missing inputs | 2a | Calculation proceeds when data are missing |
| Ambiguous terminology | 3a | Ambiguous, confusing or contradictory terminology |
|  | 3b | Ratio factors labelled incorrectly |
| Data entry issues | 4a | Limited precision prevents recording of at least 1 decimal place for measurements requiring such precision, e.g. mmol/L for blood glucose. |
|  | 4b | Problems entering or saving data in the correct input fields |
| Output issues |  |  |
| Clinical model violation † | 5a | Calculation using impossible inputs, e.g. zero-valued blood glucose |
|  | 5b | Increasing correction bolus in response to falling blood glucose |
|  | 5c | Meal bolus not offset by negative correction bolus (blood glucose below target blood glucose) |
| Formula inconsistency | 6a | App does not conform to stated formula, for any reason |
| Input-output mismatch | 7a | Output is not updated reliably if inputs are changed (Automatic calculators) |
|  | 7b | Output is not updated reliably when calculation button is pressed (Manual calculators) |
| Other software errors | 8a | Any other software issue, e.g. crash, not described above |

† There are many contextual factors which might affect the suitability of calculation, for example the effect of pregnancy on insulin resistance. Here, we considered only those factors that apply to all patients. The possible impact of contextual factors is addressed in the discussion.
