# Supplementary material for: Smartphone apps for calculating insulin dose: a systematic assessment
Source: BMC Med. 2015 May 6;13:106. doi: 10.1186/s12916-015-0314-7 (PMC4433091; doi:10.1186/s12916-015-0314-7)
Supplement: Additional file 2: — Supplementary tables AF5-AF7. Data tables. Contents: Table AF5 - Characteristics of excluded apps; Table AF6 - Basic characteristics of included apps; Table AF7 - Calculator design of included apps. [file 12916_2015_314_MOESM2_ESM.docx]

# Additional File 2

## Supplementary Methods and Tables

Supplementary Table AF5

## Characteristics of excluded apps

Supplementary Table AF6

## Basic characteristics of included apps

Supplementary Table AF7

## Calculator design of included apps

Supplementary Table AF5

**Characteristics of excluded apps**

The table below provides details of apps that were excluded after download and review and the reasons for exclusion.

| **App Name** | **Platform** | **Version** | **Developer** | **Description** | **Reason for Exclusion** † |
| --- | --- | --- | --- | --- | --- |
| BG Monitor Diabetes (Donate) | Android | 2.0 | Gordon Wong | Diabetes diary with insulin dose suggestion. | Duplicate |
| Diabetes Personal Calculator | iOS | 1.2 | iTenuto Soft | Insulin dose calculator. Lite version. | Duplicate |
| DiaLog: Diabetes Logbook Demo | Android | 1.2.3 | David Froehlich | Diabetes diary with suggested insulin dose calculator. | Duplicate |
| GliControl (Demo) | Android | 4.5.0 | softbycloud.com | Diabetes diary with separate meal dose insulin calculator. Demo version. | Duplicate |
| Glucool Diabetes | Android | 1.4.3.1 | 3qubits | Diabetes diary with separate insulin calculator. Light version. | Duplicate |
| Glucose Companion Free | iOS | 2.3 | Maxwell Software | Diabetes diary with separate insulin calculator. Free version. | Duplicate |
| Infusion Calculator | Android | 1.4 | CityJams | Collection of calculators including questionnaire-style insulin dose calculator. Standalone version. | Duplicate |
| Insulin Dose Advisor ControlWizard | iOS | 2.61 | Piet van der Wal | Meal bolus calculator. Previous version of app including in testing. | Duplicate |
| mDiab Lite | Android | 1.0 | INFOKOM Informations- und Kommunikationsgesellschaft mbH | Diabetes diary with separate insulin calculator. Lite version. | Duplicate |
| mDiab Lite | iOS | 1.1 | INFOKOM Informations- und Kommunikationsgesellschaft mbH | Diabetes diary with separate insulin calculator. Lite version. | Duplicate |
| Glucosurfer | Android | 2.6 | Glucosurfer.org | Diabetes diary. No insulin calculator. | No calculator |
| Your Personal Health | Android | 1.0.0 | Darren Gates | Medical triage assessment questionnaires. No calculators. | No calculator |
| B. Braun SGC Tutorial | iOS | 2.0 | B. Braun Melsungen AG | Tutorial for clinical insulin infusion system. No functional insulin calculator. Targeted at clinicians. | No calculator |
| Drips and Pumps | Android | 2.0 | CocoLabz | Collection of infusion rate calculators including insulin. Targeted at clinicians. | Other calculation |
| Hospital Diabetes | Android | 1.4 | David Carmody | Information about diabetes management with unit conversion calculators. Targeted at clinicians. | Other calculation |
| IV Drip Rate Professional | Android | 1.0 | geekpro | Collection of infusion rate calculators including insulin. | Other calculation |
| IV Drips | Android | 3.1 | Jonsap | Collection of infusion rate calculators including insulin. | Other calculation |
| Med Tools | Android | 1.0 | Darren Gates | Collection of medical calculators. No insulin calculator. Targeted at clinicians. | Other calculation |
| MedCalc 3000 Complete | Android | 13.3.1 | Foundation Internet Services | Collection of management algorithms and medical calculators including insulin resistance calculator. Targeted at clinicians. | Other calculation |
| MEDGuide Emergency | Android | 1.6 | JUAN C.L.C. | Emergency management algorithms including insulin infusion preparation calculator. Targeted at clinicians. | Other calculation |
| Medi Converter | Android | 1.2 | CocoLabz | Collection of unit conversion calculators. No insulin calculator. Targeted at clinicians. | Other calculation |
| Medical Calculators | Android | 1.0 | Darren Gates | Collection of medical calculators. No insulin calculator. Targeted at clinicians. | Other calculation |
| MedicCalc Paramedic Calculator | Android | 1.1 | LINC Mobile Applications | Collection of medical calculators. No insulin calculator. Targeted at clinicians. | Other calculation |
| Nurse's Toolbox | Android | 1.0.1 | Medical Wizards, LLC | Collection of management algorithms and medical calculators including pediatric insulin infusion calculator. Targeted at clinicians. | Other calculation |
| OmniMedix Medical Calculator | Android | 3.0 | Omnimedic Solutions | Collection of medical calculators. No insulin calculation. Targeted at clinicians. | Other calculation |
| Paramedic Meds | Android | 3.1 | Jonsap | Collection of medical calculators including insulin infusion preparation calculator. | Other calculation |
| Daily Carb - Carbohydrate, Glucose, Medication, Blood Pressure and Exercise Tracker | iOS | 1.7 | Maxwell Software | Food and weight diary with BMI calculator. | Other calculation |
| Hypo | iOS | 1.1 | Philippe Mougin | Glucose to offset hypoglycemia calculator. | Other calculation |
| MedCalc 3000 Endocrine | iOS | 9.1 | Foundation Internet Services, LLC | Collection of management algorithms and medical calculators including insulin resistance calculator. Targeted at clinicians. | Other calculation |
| Skyscape Medical Resources | iOS | 1.18.25 | Skyscape | Medical information including medical calculators. No insulin calculation. Targeted at clinicians. | Other calculation |
| BS3 Basic Tools Pack | Android | 1.0 | ABCGO s.r.l. | Collection of calculators including insulin required to offset glucose bolus calculator. Targeted at clinicians. | Other insulin calculation |
| DiAppBetes | Android | 1.0 | Horizon Strategic Partners Ltd | Insulin prescribing and hypoglycemia management information. Includes insulin initiation and total dose adjustment calculators. Targeted at clinicians. | Other insulin calculation |
| Insulin Quantity Calculator | Android | 1.1 | DunnRight Software | Insulin consumption calculator. | Other insulin calculation |
| Insulin Therapy | Android | 1.0 | Prestaciones Médicas RCCC | Insulin initiation and dose adjustment calculators. | Other insulin calculation |
| Smart e-SMBG - Diabetes | Android | 1.1.1 | ARKRAY, Inc. | Diabetes diary with carbohydrate:insulin and insulin sensitivity calculators. | Other insulin calculation |
| BasalCalc | iOS | 1.1.0 | Guilherme de Paula | Insulin pump initiation basal rate calculator. | Other insulin calculation |
| BG to Insulin (Correction) Factor Calculator | iOS | 1.1.0 | Guilherme de Paula | Insulin sensitivity calculator. | Other insulin calculation |
| Diabetes Carb/Ins RatioWizard PRO | iOS | 1.2 | Piet van der Wal | Carbohydrate:insulin ratio calculator. | Other insulin calculation |
| Diagnosis and Management of Gestational Diabetes | iOS | 1.1 | Greggory DeVore | Information about management of gestational diabetes. Includes insulin initiation calculator. Targeted at clinicians. | Other insulin calculation |
| DiAppBetes | iOS | 1.1 | Horizon Strategic Partners Ltd | Insulin prescribing and hypoglycaemia management information. Includes insulin initiation and total dose adjustment calculators. Targeted at clinicians. | Other insulin calculation |
| EZ Insulin Calculator | iOS | 1.02 | Crystal Clear Solutions | Insulin pump initiation basal rate calculator. | Other insulin calculation |
| Glucontrol | iOS | 1.01 | hans-peter van leeuwen | Insulin infusion rate adjustment calculator. Targeted at clinicians. | Other insulin calculation |
| Insulin Dosing Algorithm | iOS | 1.6 | Austin Physician Productivity, LLC | Insulin initiation and dose adjustment calculators. | Other insulin calculation |
| Insulin TDD and BMI Calculator | iOS | 1.1.0 | Guilherme de Paula | Insulin total dose and BMI calculators. | Other insulin calculation |
| Insulin To Carb (I:C) Ratio Calculator | iOS | 1.1.0 | Guilherme de Paula | Insulin:carbohydrate ratio calculator. | Other insulin calculation |
| Insulin-On-Board (IOB) Calculator | iOS | 1.0.0 | Guilherme de Paula | Insulin on board calculator. | Other insulin calculation |
| SugarPal Diabetes Manager | iOS | 1.0 | L F Tiemeijer | Basal dose calculator. | Other insulin calculation |
| NutriPlus-Sweet Control | Android | 2.8 | ReFleX Wireless | Diabetes diary with insulin calculator. Requires device to use. | Requires device |

† Reasons for exclusion: Other insulin calculation = the app performed some calculation relating to insulin administration other than rapid acting insulin dosing, for example calculating the amount of insulin to add to an intravenous infusion set. Other calculation = the app performed one or more calculations but they were unrelated to insulin.

Supplementary Table AF6

**Basic characteristics of included apps**

| **App ID** † | **App Name** | **Version** | **Developer** | **Region (Country)** | **iOS** | **Android** | **App Type** |
| --- | --- | --- | --- | --- | --- | --- | --- |
| 5194 | BG Monitor Diabetes | 6.2 | Gordon Wong | North America (USA) | No | Yes | Diary with Suggested Dose |
| 1140 | Bolus | 1.0 | Krunoslav Djakovic | Europe (Serbia) | Yes | Yes | Standalone Calculator |
| 1086 | BolusCalc | 1.0.0 | Guilherme de Paula | North America (USA) | Yes | No | Standalone Calculator |
| 4037 | D Sharp Diabetes | 1.0 | D Sharp Diabetes | North America (Canada) | No | Yes | Diary with Suggested Dose |
| 5309 | D.O.T. Beta (Insulin Calc) | 1.2.1 | VoidFlinger | North America (USA) | No | Yes | Standalone Calculator |
| 1223 | DAFNE Online | 1.6.2 | Simon Fisher | Europe (UK) | Yes | No | Diary with Suggested Dose |
| 949 | Diabetes 360 Lite | 1.6.4 | Joseph DiMaggio | North America (USA) | Yes | No | Diary with Suggested Dose |
| 1023 | Diabetes Manager | 1.3 | iTenuto Soft | North America (USA) | Yes | No | Calculator with Log |
| 5310 | Diabetes Tracker | 1.34 | Daniel Development | Australasia (Indonesia) | No | Yes | Diary with Suggested Dose |
| 4465 | Diabetic Dosage Calculator | 1.1 | Kalianne Neumann | North America (USA) | Yes | Yes | Standalone Calculator |
| 5468 | DIABETooL Insulin Calc | 1.3.9.1 | Hornetbzz | Europe (France) | No | Yes | Calculator with Log |
| 5269 | DiaLog: Diabetes Logbook | 1.2.3 | David Froehlich | Europe (Germany) | No | Yes | Diary with Suggested Dose |
| 1041 | Diamedic | 3.1.1 | Nicholas Martin | North America (USA) | Yes | No | Diary with Separate Calculator |
| 1205 | DM Diary | 1.7 | Ingemar Jacob | Europe (Germany) | Yes | No | Calculator with Log |
| 1120 | GliControl | 4.2 | Hugo Ferreira | Europe (Portugal) | Yes | Yes | Diary with Separate Calculator |
| 4608 | Glucool Diabetes Premium | 1.4.3.1 | 3qubits | Europe (France) | No | Yes | Diary with Separate Calculator |
| 1045 | Glucose Companion | 2.3 | Maxwell Software | Asia (China) | Yes | No | Diary with Separate Calculator |
| 4175 | Glucose Meter | 1.6.3 | Francisco J Belchi | Europe (UK) | No | Yes | Diary with Separate Calculator |
| 1005 | HelpDiabetes | 2.0.5 | Johan Degraeve | Europe (Netherlands) | Yes | Yes | Diary with Suggested Dose |
| 975 | iBolusCalc - Diabetes Blood Glucose Helper | 1.12 | effective delivery, inc. | North America (USA) | Yes | No | Standalone Calculator |
| 1097 | iCare-D | 1.0.3 | Pyeonghwa Uhealth Co. Ltd. | Asia (Korea) | Yes | Yes | Diary with Separate Calculator |
| 5543 | Insulin Bolus Calculator | 1.3 | Felix Software Company Limited | Asia (Hong Kong) | No | Yes | Calculator with Log |
| 1047 | Insulin Calc | 1.0 | Maxwell Software | Asia (China) | Yes | No | Standalone Calculator |
| 1017 | Insulin Calculator | 1.0 | Jagdeep Nagpal | Europe (UK) | Yes | Yes | Standalone Calculator |
| 987 | Insulin Calculator | 1.3.2 | Chris Bowley | Europe (UK) | Yes | No | Standalone Calculator |
| 1099 | Insulin Dose Advisor ControlWizard-2 | 3.1 | Knowledge is Power Solutions | Europe (Netherlands) | Yes | Yes | Diary with Suggested Dose |
| 3908 | Insulin Dose Calculator | 1.0 | CityJams | North America (USA) | No | Yes | Standalone Calculator |
| 1123 | Insulin Dose Calculator Pro | 1.2 | STRUCTIVA | Europe (Sweden) | Yes | No | Standalone Calculator |
| 3538 | Insulin Unit Calculator | 2.0 | Jason Timm | North America (USA) | No | Yes | Standalone Calculator |
| 1193 | Insulin Units | 1.2 | Kenneth Wallace | North America (USA) | Yes | No | Standalone Calculator |
| 1177 | InsulinPlus | 1.0.2 | SquareMed Software GmbH | Europe (Germany) | Yes | No | Standalone Calculator |
| 1015 | mDiab | 1.1 | INFOKOM Informations- und Kommunikationsgesellschaft mbH | Europe (Germany) | Yes | Yes | Diary with Separate Calculator |
| 4666 | My Diabetes | 1.3.4 | Rossen Varbanov | Europe (Bulgaria) | No | Yes | Diary with Separate Calculator |
| 1175 | myBolus | 2.1.0 | Christian Schurk | Europe (Germany) | Yes | No | Standalone Calculator |
| 1179 | Pumps4kids | 1.2 | Debbie McCann | Australasia (Australia) | Yes | Yes | Information and Separate Calculator |
| 990 | RapidCalc Diabetes Manager | 1.4.1 | Gilport Enterprises Pty Ltd | Australasia (Australia) | Yes | No | Calculator with Log |
| 941 | Track3 - Diabetes Planner, Logbook and Carb Counter (SI version) | 4.5 | Coheso, Inc | North America (USA) | Yes | No | Diary with Separate Calculator |

† A unique ID assigned to the app for the study, can be used to reconcile apps between tables AF6 and 7.

Supplementary Table AF7

**Calculator design of included apps**

|  | **Inputs** | | | | | | **Outputs** | | | |
| --- | --- | --- | --- | --- | --- | --- | --- | --- | --- | --- |
| **App ID** | **Method** | **Glucose Units** | **Glucose Adjustment Factor ^a^** | **Carb Units** | **Carb Adjustment Factor ^b^** | **Additional Inputs** | **Meal Bolus ^c^** | **Correction Bolus ^d^** | **Corrected Meal Bolus ^e^** | **IOB Compensation ^f^** |
| 941 | Manual | mmol/L | BG:I | grams | I:C | * | Yes | Yes (±) | Yes | No |
| 949 | Manual | mg/dL, mmol/L | BG:I | Carbs | I:C | * | Yes | Yes (±) | Yes | No |
| 975 | Manual | mg/dL | BG:I | Carbs | I:C | Activity Levels | Yes | Yes (±) | Yes | Yes |
| 987 | Manual | mg/dL, mmol/L | I:BG | grams | I:C | * | Yes | Yes (±) | Yes | No |
| 990 | Manual | mg/dL, mmol/L | BG:I | grams, 10g portions, 12g portions (bread units), 15g portions | Fixed | Activity Levels | Yes | Yes (±) | Yes | Yes |
| 1005 | Manual | * | * | grams | I:C | * | No | Yes (+) | No | No |
| 1015 | Manual | * | * | 12g portions | Fixed | * | No | Yes (+) | No | No |
| 1017 | Manual | mmol/L | I:BG | grams | Fixed | * | Yes | Yes (±) | Yes | No |
| 1023 | Manual | mg/dL, mmol/L | BG:I | Carbs | I:C | * | Yes | Yes (±) | Yes | No |
| 1041 | Manual | mg/dL, mmol/L | BG:I | grams | I:C | * | Yes | Yes (+) | Yes | No |
| 1045 | Manual | mg/dL, mmol/L | BG:I | grams | I:C | * | Yes | Yes (±) | Yes | No |
| 1047 | Manual | mg/dL, mmol/L | BG:I | grams | I:C | * | Yes | Yes (±) | Yes | No |
| 1086 | Manual | mg/dL | I:BG | grams | I:C | Activity Levels | Yes | Yes (±) | Yes | Yes |
| 1097 | Manual | mg/dL, mmol/L | I:BG | grams | I:C | * | Yes | Yes (±) | Yes | No |
| 1099 | Manual, Learned^g^ | * | * | Carbs | I:C | * | No | Yes (+) | No | No |
| 1120 | Manual | * | * | grams | I:C | * | No | Yes (+) | No | No |
| 1123 | Manual | mg/dL, mmol/L | BG:I | grams, exchange units | I:C | Activity Levels | Yes | Yes (±) | Yes | Yes |
| 1140 | Manual | (Unit Agnostic) | BG:I | grams | I:C | Ketones Present | Yes | Yes (±) | Yes | No |
| 1175 | Manual | mg/dL, mmol/L | I:BG | BU (user customisable grams/BU) | Fixed | * | Yes | Yes (±) | Yes | No |
| 1177 | Manual | mg/dL, mmol/L | BG:I | BE, KE, portions | I:C | * | Yes | Yes (±) | Yes | No |
| 1179 | Manual | mg/dL, mmol/L | BG:I | (Unit Agnostic) | I:C | * | Yes | Yes (±) | Yes | No |
| 1193 | Manual | mg/dL | BG:I | grams | I:C | * | Yes | Yes (+) | Yes | No |
| 1205 | Manual | mg/dL, mmol/L | BG:I | KE, BE, KH | Fixed | * | Yes | Yes (+) | Yes | No |
| 1223 | Manual | mmol/L | BG:I | CP | Flexible | Activity Levels | Yes | Yes (±) | No | Yes |
| 3538 | Manual | mg/dL | BG:I | grams | I:C | * | Yes | Yes (±) | Yes | No |
| 3908 | Manual | (Unit Agnostic) | I:BG | grams | I:C | Activity Levels | Yes | Yes (±) | Yes | Yes |
| 4037 | Manual | mg/dL, mmol/L | I:BG | grams | I:C | * | Yes | Yes (±) | Yes | No |
| 4175 | Manual, Bluetooth^h^ | mg/dL, mmol/L | BG:I | Carbs | I:C | Activity Levels | Yes | Yes (±) | Yes | Yes |
| 4465 | Manual | mg/dL, mmol/L | BG:I | (Unit Agnostic) | Fixed | * | Yes | Yes (±) | Yes | No |
| 4608 | Manual | mg/dL, mmol/L | BG:I | Carbs | I:C | Activity Levels | Yes | Yes (±) | Yes | Yes |
| 4666 | Manual | mg/dL, mmol/L | BG:I | grams | I:C | * | Yes | Yes (±) | Yes | No |
| 5194 | Manual | mg/dL, mmol/L | BG:I | grams | I:C | * | Yes | Yes (+) | Yes | No |
| 5269 | Manual | mg/dL, mmol/L | BG:I | 10g portions, 12g portions (bread units) | Fixed | Activity Levels | Yes | Yes (±) | Yes | No |
| 5309 | Manual | (Unit Agnostic) | BG:I | Carbs | Flexible | * | Yes | Yes (+) | Yes | No |
| 5310 | Manual | (Unit Agnostic) | BG:I | Carbs | Fixed | * | Yes | Yes (+) | Yes | No |
| 5468 | Manual | mg/dL, mmol/L | BG:I | grams | C:I | Lipid Intake, Activity Levels | Yes | Yes (±) | Yes | Yes |
| 5543 | Manual | mg/dL, mmol/L | BG:I | grams, 15g portions (exchanges) | I:C | * | Yes | Yes (+) | Yes | No |

Abbreviations: Carb = carbohydrate

^a^ BG:I = blood glucose to insulin ratio (Insulin sensitivity), I:BG = blood glucose to insulin ratio.

^b^ C:I = Carbohydrate to insulin ratio (Carbohydrate factor), I:C = insulin to carbohydrate ratio.

^c^ Meal Bolus = Dose of rapid acting insulin calculated to offset planned or actual carbohydrate intake.

^d^ Correction Bolus = Dose of rapid acting insulin calculated to offset difference in measured and target blood glucose. Symbol in parentheses indicates if correction boluses only calculated when blood glucose exceeds target value (+) or if negative values are also allowed (±). A negative correction bolus arises when blood glucose is below target value and indicates that any meal bolus should be reduced accordingly.

^e^ Corrected Meal Bolus = The total dose calculated as the sum of meal and correction boluses.

^f^ IOB = Insulin on Board, If Yes indicates that the app adjusts any dose according to insulin that has previously been administered but not yet fully metabolized.

^g^ This app learned carbohydrate adjustment factor using 2-hour post-meal glucose measurements to measure the response to administered insulin.

^h^ This app supported automatic transfer of glucose readings from a Bluetooth-enabled glucose meter.
